# Psychological and contextual risk factors for first‐onset depression among adolescents and young people around the globe: A systematic review and meta‐analysis

**DOI:** 10.1111/eip.13300

**Published:** 2022-04-07

**Authors:** Gloria A. Pedersen, Crystal Lam, Megan Hoffmann, Zuzanna Zajkowska, Annabel Walsh, Christian Kieling, Valeria Mondelli, Helen L. Fisher, Kamal Gautam, Brandon A. Kohrt

**Affiliations:** ^1^ Division of Global Mental Health, Department of Psychiatry, School of Medicine and Health Sciences The George Washington University Washington District of Columbia USA; ^2^ Department of Psychological Medicine, Institute of Psychiatry, Psychology & Neuroscience King's College London London UK; ^3^ Child & Adolescent Psychiatry Division, Department of Psychiatry, Hospital de Clínicas de Porto Alegre Universidade Federal do Rio Grande do Sul Porto Alegre Brazil; ^4^ National Institute for Health Research (NIHR) Maudsley Biomedical Research Centre South London and Maudsley NHS Foundation Trust, King's College London London UK; ^5^ Social, Genetic & Developmental Psychiatry Centre, Institute of Psychiatry, Psychology & Neuroscience King's College London London UK; ^6^ ESRC Centre for Society and Mental Health King's College London London UK; ^7^ Transcultural Psychosocial Organization Nepal (TPO Nepal) Kathmandu Nepal

**Keywords:** adolescent, developing countries, early diagnosis, risk assessment, risk factors

## Abstract

**Aim:**

Identifying predictors for future onset of depression is crucial to effectively developing preventive interventions. We conducted a systematic review and meta‐analysis to identify risk factors for first‐onset depression among adolescents and young people.

**Methods:**

We searched MEDLINE (Ovid), PsycINFO, Cochrane Database, Web of Science, Lilacs, African Journals Online and Global Health (July 2009 to December 2020) for longitudinal studies assessing risk factors for first‐onset depression among adolescents and young people aged 10–25 years. Meta‐analyses generated summary odds ratio (OR) estimates. Registration: PROSPERO CRD42018103973.

**Results:**

Nineteen studies representing 21 unique populations were included in the meta‐analysis. Among studies reporting race/ethnicity, 79% of participants were of White/European descent. Seventeen studies were from high‐income countries, with only two from an upper‐middle‐income country (China). Odds for first‐onset depression were significantly greater for girls compared to boys (*n* = 13; OR = 1.78 [1.78, 2.28], *p* < 0.001) and for youth with other mental health problems at baseline (*n* = 4; OR = 3.20 [1.95, 5.23], *p* < 0.001). There were non‐significant associations for negative family environment (*n* = 8; OR = 1.60 [0.82, 3.10], *p* = 0.16) and parental depression (*n* = 3; OR = 2.30 [0.73, 7.24], *p* = 0.16).

**Conclusions:**

Most longitudinal studies do not report risk factors specifically for first‐onset depression. Moreover, predictive data are limited to predominantly White populations in high‐income countries. Future research must be more ethnically and geographically representative. Recommendations are provided for consistent and comprehensive reporting of study designs and analyses of risk factors for first‐onset depression.

## 1 INTRODUCTION

Due to its early‐onset and chronicity, depression is a leading contributor to disability‐adjusted life‐years (Whiteford et al., 2013). Neglecting to prevent and treat early‐life onset of depression has severe consequences: in 2019, global rates of suicide are highest between ages 20 and 25 years, and most adolescents who died by suicide (88%) were from low‐and‐middle‐income countries (LMIC) (World Health Organization, 2019). This statistic is particularly arresting considering that 90% of the world's adolescents live in LMICs, yet research identifying predictors of depression conducted in these settings is lacking (Kieling et al., 2011). Previous systematic reviews and meta‐analyses have been conducted on risk factors for adolescent depression, but the overall generalizability of the evidence is limited. Many reviews involve a restricted scope of risk factors (Cairns et al., 2014; Kohler et al., 2018), are limited to Western Educated Industrialized Rich Democratic (WEIRD) societies (e.g., North America, Western Europe) (Bor et al., 2014; Cairns et al., 2014; Reiss, 2013), and often define adolescents with a narrow age range (≤19 years) (Bor et al., 2014; Braithwaite et al., 2017; Cairns et al., 2014; Dardas et al., 2018; Koenig et al., 2016; Polanczyk et al., 2015; Reiss, 2013; Sanger et al., 2015; Stirling et al., 2015; Vilgis et al., 2015; Yap et al., 2014).

Identification of a broad scope of psychological and contextual risk factors across settings offers the potential to accurately assess risk for first‐onset depression and more reliably inform prevention strategies and mechanisms of action (Cuijpers et al., 2012; Kieling et al., 2019). The World Health Organization and the United Nations agree that a wide age range for adolescence (<25 years) is pivotal, particularly in LMICs, to account for contextual factors that may impact the development of the adolescent brain transitioning into adulthood across cultures (United Nations Department of Economic and Social Affairs, 2010; WHO, 2011). These recommendations corroborate research showing that brain maturation in adolescence is defined between ages 10 and 24 years (Arain et al., 2013), with experts insisting that this more inclusive age range is required to account for major developmental impacts framed by social policies, service systems and laws (Sawyer et al., 2018). Accounting for a range of risk factors also increases the proficiency of existing predictive models that can be evaluated in high‐income country (HIC) and LMIC cohorts.

We found nine previous systematic reviews of risk factors for adolescent depression. One review identified 113 studies presenting only modifiable risk and protective factors (e.g., substance use, dieting, negative coping strategies and weight) shown to influence development of adolescent depression (Cairns et al., 2014). Of the 113 studies, only 3% represented populations in LMICs. In another review, modifiable predictors of depression following maltreatment (e.g., physical or sexual abuse) were identified for adolescents <18 years across 22 studies; though authors reported a lack of well‐designed prospective studies (Braithwaite et al., 2017). Two reviews identified 76 studies presenting socioeconomic risk factors (community safety and ethnic minority status) (Reiss, 2013; Stirling et al., 2015), though 90% of the studies represented high‐and‐upper‐middle‐income populations, and neither review focused on prospective effects for depression onset. Another review identified effects for parental factors increasing risk for anxiety and depression in adolescent offspring aged 12–18 (Yap et al., 2014), though results were combined from cross‐sectional and prospective studies. All reviews were limited to a 19‐year age range. No identified reviews emphasized prospective longitudinal studies evaluating a range of contextual and psychological risk factors among adolescents ≤25 years across universal settings. Therefore, we conducted a meta‐analysis of psychological and contextual risk factors predicting first‐onset depression among adolescents ≤25 years in the global literature.

## 2 METHODS

This systematic review and meta‐analysis followed the Preferred Reporting Items for Systematic Review and Meta‐Analysis (PRISMA) 2015 checklist (PRISMA‐P Group et al., 2015). A systematic review protocol for the evaluation of risk factors associated with depression among adolescents and young people was registered in PROSPERO (https://www.crd.york.ac.uk/prospero/#myprospero CRD42018103973) and published (Pedersen et al., 2019). The current review and meta‐analyses in this article reflect Objective #1 of the published protocol. Of note, for the purposes of simplicity when describing the methods and results, we have collapsed ‘adolescent’ and ‘young people’ to use of the term ‘young people’ as being inclusive of both adolescents and young people.

### 2.1 Study eligibility

We searched MEDLINE (via Ovid), PsycINFO, Cochrane Database of Systematic Reviews, Web of Science (Core Collection), LILACS, African Journals Online, and Global Health for longitudinal studies assessing risk factors for first‐onset depression in young people aged 10–25 in HICs and LMICs. HIC and LMIC were defined using World Bank criteria (The World Bank, 2018). For any country that changed classification during longitudinal data collection, we referred to the World Bank criteria for that country at the time the cohort was initiated. Further information on definitions and classifications used throughout this study can be found in the study protocol (Pedersen et al., 2019).

The searches were conducted in English, though language was not used to limit the search output. The LILACS and African Journals Online databases were specifically included to ensure coverage of Latin‐American, the Caribbean, and African peer‐reviewed published literature. Our team included translators for Portuguese, Italian, Nepali and Mandarin languages; if other languages needed translation, we consulted the IDEA consortium network and reference librarians at the authors' universities as first and second resources, respectively, to identify a translator.

We initially conducted the search according to the protocol strategy, which did not put a limit on the date of publication. However, given the large number of results, we altered the inclusion criteria to include the 10 years prior to the search (January 1, 2009 to December 31, 2019). We considered this representative because the median year for all publications on the topic was 2012, for example, 50% of all publications meeting inclusion criteria happened after 2012. The search was later extended through December 17, 2020, to be more inclusive of up‐to‐date findings. The resulting time period was 12 years from 2009 through 2020. See Box S1 in supplementary materials for a sample search strategy for Medline (Ovid).

### 2.2 Study selection

The database results were exported to EndNote X8, a reference management system that was also used to remove duplicates, resulting in 12 753 articles titles and abstracts for review. Using a charting form in Google sheets, four reviewers screened the same title and abstracts in batches of 10 articles from the EndNote export results to assess inter‐rater reliability (IRR) on inclusion/exclusion criteria for title and abstract screening, with any discrepancies resolved by a fifth reviewer. The reviewers repeated this process with subsequent batches of 10 articles, recording all final agreements per article. Upon the fifth batch of 10 articles, a blinded 95% IRR was reached. After achieving this IRR, the remaining titles and abstracts (*n* = 12, 703) were then divided among the reviewers for independent screening with discussions and stages of IRR reassessment (*see* Figure S1 in supplementary file for details on the title and abstract screening and IRR processes). After the 12 753 titles and abstracts were screened, 6394 titles and abstracts were left for potential inclusion, which then underwent a re‐screening. IRR was reassessed at this time following the same procedure as described above (e.g., all reviewers screening batches of 10 until an IRR of 95% was achieved). The IRR of 95% was achieved in the first batch of 10, therefore the remaining 6384 articles were re‐screened independently by reviewers. This resulted in a final 496 articles included for the full‐text screening stage. For full‐text screening, the same three reviewers assessed IRR for screening full‐text articles until a blinded 90% agreement was reached (at *n* = 3 articles), and then independently assessed the remaining 493 articles for eligibility. Justification for excluding full‐text articles was recorded for each study, and a fourth reviewer resolved any disagreements between the three reviewers.

### 2.3 Eligibility criteria

Eligibility criteria were specified according to the Population, Exposure, Comparison, Outcome (PIECO) criteria and in‐line with PRISMA guidelines. Table 1 reviews the inclusion and exclusion criteria. Studies using prospective longitudinal designs published between January 1, 2009 and December 17, 2020, were included. Discussion papers, letters, editorials, case studies or case series and qualitative studies without a quantitative element were not included. Studies were excluded if the prospective time point for depression measurement was <6 months. Studies were excluded if history of major depressive disorder (MDD) and/or MDD at baseline was not controlled for in the analysis. During full‐text screening, reviewers used an additional screening chart to identify first‐onset depression (Suppl. Table S1). Where analysis of first‐onset depression was unclear (e.g., baseline or history of MDD was not reported or first‐onset was combined with previous onset at follow‐up), but all other criteria were met, we contacted authors by email for clarification. Contextual and psychological risk factors for first‐onset depression were included. After full‐text screening was completed, but prior to conducting the meta‐analysis, we created risk factor subgroups a priori by thematically grouping similar risk factors into categories following guidance by the IDEA consortium experts and existing predictive models for adolescent depression (Wahid et al., 2021). For performing meta‐analysis of randomized controlled trials (RCT), Cochrane recommends ‘Two studies is a sufficient number to perform a meta‐analysis, provided that those two studies can be meaningfully pooled and provided their results are sufficiently “similar”,’ (Ryan, 2016) with the basis of being ‘sufficiently similar’ depending on whether the studies are fairly homogeneous and lower risk of bias. Given observational cohort study designs introduce more bias overall than RCT designs, and because this study did not limit inclusion criteria to a single tool for measuring depression, nor a single context, the authors of this review agreed that a more robust analysis would require at least three or more studies per thematic risk factor category in order to be included in the meta‐analysis.

**TABLE 1:** Inclusion and exclusion criteria for prospective longitudinal studies examining risk factors for first‐onset depression in young people

Population	Exposure	Comparison	Outcome
*Inclusion criteria*			
Any adolescents evaluated longitudinally <25 years at baseline	Risk and protective factors	Adolescents who do not develop depression during assessed time periods of adolescence and young adult development; as well as the adolescent as her/his own control for premorbid assessment Longitudinal/prospective study	At least one time point of prospectively measured first onset of depression or depressive symptoms through a categorical diagnostic interview or continuous measure with cut‐off score (e.g., self, parent, and/or teacher report) prior to age 25 Can include co‐morbidity

*Exclusion criteria*			
Persons <25 years at study baseline Persons limited to only specific medical subpopulations (e.g., only young people with HIV, diabetes, intellectual disabilities, pregnant women)	Experimental stressful exposures	Not longitudinal prospective study (e.g., cross‐sectional, reviews, discussion papers, non‐research, letters, editorials, case studies or case series, animal studies, qualitative studies without a quantitative element) Only retrospective data Not observational (intervention/prevention trial) No measurement of depression between ages 10 and 24 years	No depression or depressive symptoms measured/reported Continuous measures without cut‐off score No quantitative depression prevalence data reported Reported follow‐up period of <6 months Lack of analysis for novel onset of depression^a^

^a^ Lack of analysis of first‐onset depression included studies that met any of the following criteria: Baseline assessment of current or prior major depressive disorder (MDD) not reported; Baseline assessment of current or prior MDD combined with first‐onset at follow‐up outcome reporting; Analysis of any disorder first‐onset (not specific to MDD) combined with first‐onset depression.

### 2.4 Outcomes

The primary outcome was first‐onset depression among young people ages 10–25 years in any population, for example, high‐, middle‐ and low‐income countries. At baseline, the age of young people should have been <25 years, with first‐onset depression reported as a dichotomous outcome and measured ≥6 months from baseline. Depression could include self‐ and adult‐informant reports with a cut‐off score, structured observations in clinical settings, and/or clinical records. Additionally, we included mixed population studies if outcomes were reported separately for the subgroup who did not have current or prior depression at baseline or if risk factors were reported controlling for baseline depression status.

### 2.5 Data extraction and analysis

For the extraction stage, a data extraction sheet for descriptive and quantitative data was developed in Google sheets with pre‐determined fields (Pedersen et al., 2019) and was approved by the IDEA consortium. Three reviewers conducted an IRR process where each reviewer extracted information for all categories for the same article. Each author was blind to each‐other's extracted content to ensure rich discussion and assess unique discrepancies across reviewers. The extraction reviewers reached a 95% IRR on the third full‐text article. Two reviewers extracted data for the remaining included studies. A third reviewer validated extraction for all studies, using weekly meetings to discuss discrepancies, update extractions and refer to a fourth reviewer if any discrepancy could not be resolved.

We assessed the quality of evidence for each study using the Systematic Assessment of Quality in Observational Research (SAQOR) (Ross et al., 2011) with converted modified Grading of Recommendations, Assessment, Development and Evaluations (GRADE) rankings (Guyatt et al., 2011; Ross et al., 2011). Reviewers discussed and piloted application of SAQOR criteria with three articles. A Google doc was created to specify discussion points and charting information for reviewers' reference during assessment of studies. After agreement was reached, two reviewers assessed the quality of the included articles, and the third reviewer validated the SAQOR criteria per article to ensure accuracy.

To assess the effect of risk factors predicting first‐onset depression, we did random‐effects meta‐analyses in Cochrane Review Manager version 5.3 (The Cochrane Collaboration, 2014). We calculated odds ratios (OR) for binary outcomes with 95% confidence intervals and two‐sided *p*‐values for each risk factor category evaluated in three or more studies. When applicable, we translated standardized mean differences (SMDs) for continuous outcomes into ORs using Campbell Collaboration's Practical Meta‐Analysis Effect Size Calculator (Campbell Collaboration, 2001) per study per risk category. Heterogeneity was assessed using the I‐squared statistic within Cochrane Review Manager 5.3 (The Cochrane Collaboration, 2014) applying ‘low, moderate, and high’ heterogeneity at the *I* ^2^ thresholds of 25%, 50%, and 75% (Higgins et al., 2003).

## 3 RESULTS

**FIGURE 1:**
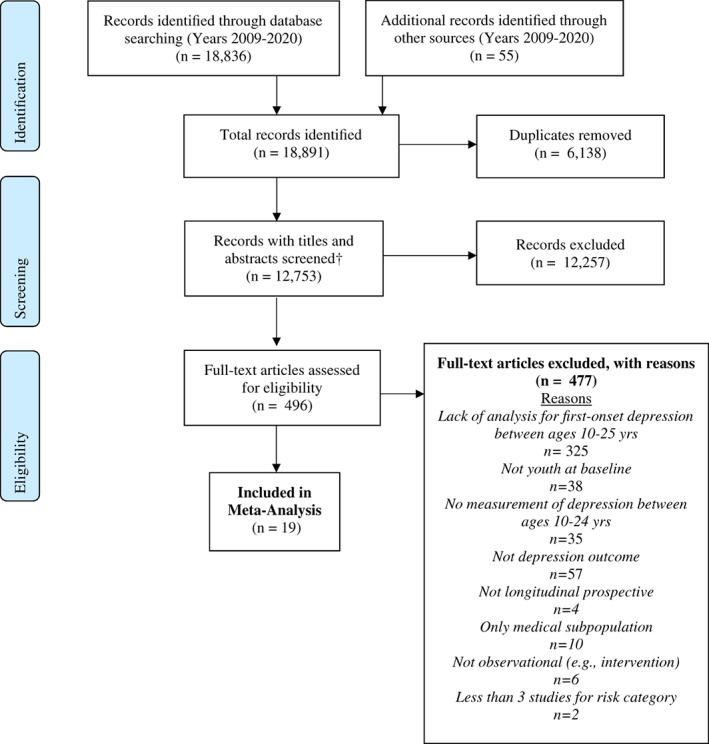
PRISMA flow diagram of study selection process. PRISMA flow diagram showing the study selection process for the systematic review and meta‐analysis. Study selection stages include (1) Identification: Number of records identified through the search across databases, additional sources identified through hand searches, and number of duplicates removed before screening. (2) Screening: Included number of titles and abstracts screened (12 years, 2009–2020) and records excluded; and (3) Eligibility: full text articles found eligible for full‐text screening, records excluded with reasons, and final number of studies included in the meta‐analysis. *Inclusive of first round screening of 12 753 titles and abstracts with four reviewers, and second round of re‐screening for 6394 titles and abstracts with three reviewers

Of the 27 803 articles from the research output, 12 753 titles and abstracts were screened of which 6394 titles and abstracts were rescreened, resulting in 496 full‐text articles screened, with a final 19 papers included in the meta‐analysis. During full‐text screening, 325 (66%) were excluded because of lack of reporting for first‐onset depression. Typical reasons for not meeting our depression outcome criteria included not controlling for depression at baseline, combining results for multiple age ranges including ages above 25, combining depression outcomes at follow‐up for those with and without a history of depression, or presenting outcome data on age ranges outside of 10–25 years. All exclusion criteria, including for the other 152 articles at full‐text screening, can be found in the PRISMA diagram in Figure 1.

Each of the 19 articles included in the meta‐analysis selected its population nationally, with one article reporting a national sample stratified by three ethnic groups (Smith et al., 2015). Therefore, we analysed 21 unique populations across risk factor categories in the meta‐analysis. Despite including multiple languages and LMIC in the search, the only studies meeting inclusion criteria were published in English, and 17 studies represented high‐income countries (United States, Canada, Australia, Germany, France, Ireland, New Zealand, the Netherlands, and United Kingdom) (Abela & Hankin, 2011; Alloy et al., 2006; Bress et al., 2013; Callaghan et al., 2017; Frey et al., 2020; Hammen et al., 2008; Hammerton et al., 2013; LeMoult et al., 2015; Little et al., 2015; Meinzer et al., 2013; Ramrakha et al., 2013; Schmid et al., 2011; Smith et al., 2015; Stavrakakis et al., 2013; Stringaris et al., 2015; Wilkinson et al., 2013; Wilkinson et al., 2018) and two studies represented an UMIC (China) (Li et al., 2018; Wu et al., 2017). Of the 19 studies, nine (47%) included information on race/ethnicity. Among all the participants (*n* = 4619) across these nine studies, 79% were White/European descent. If all participants for the China‐based studies are assumed to be East Asian, then among these participants (11 studies, *n* = 9242), approximately 39% are White/European descent, and 50% are East Asian descent. The studies are summarized in Table 2.

**TABLE 2:** Description of included studies (*n* = 19) examining first‐onset depression in young people

First author (year)	Country (HIC, UMIC, LMIC)^a^	Total baseline sample size	Sample: % White/European descent	Baseline sample size: non‐depressed	Baseline age range (mean age) [SD]	Length of follow‐up	Sample size: Follow‐up with first onset depression/total	Depression instrument^b^
Abela (2011)	Canada, USA (HIC)	382	71%	314	11–15 years (12.58 years) [1.09]	2 years	MDD history controlled for in analysis	K‐SADS
Alloy (2006)	USA (HIC)	185	78%	185	19 years	2.5 years	49/185	BDI, SADS‐L
Bress (2013)	United States (HIC)	84	97%	68	15–17 years	2 years	16/68	PHQ‐9, DISC
Callaghan (2017)	Australia (HIC)	115	Not reported	115	(12.47 years)	5.5‡ years	14/101	K‐SADS
Frey (2020)	Germany (HIC)	61 199	Not reported	60 066	6 years	13 years	6156/60066	ICD‐10
Hammen (2008)	Australia (HIC)	699	Not reported	600	15 years	5 years	131/600	K‐SADS; SCID
Hammerton (2013)	United Kingdom (HIC)	337	Not reported	281	9–17 years (12.4 years)	2.39 years^c^	24/281	CAPA
LeMoult (2015)	USA (HIC)	62	71%	62	(12.31 years)	5.69 years^c^	33/62	K‐SADS; SCID
Li (2018)	China (UMIC)	4237	0%	3196	(13.9 years) [.7]	9 months	515/3196	CES‐D
Little (2015)	Australia (HIC)	174	86%	174	11.4–13.7 years (12.7 years)	6 years	36/137	K‐SADS
Meinzer (2013)	USA (HIC)	1507	92%	1222	16 years [1.2]	8 years^c^	309/1222	K‐SADS
Smith (2015)	UK (HIC)	337; 249; 380 (966 total)	39%	Not reported	11–12 years	2 years	MDD history controlled for in analysis	SMFQ
Ramrakha (2013)	New Zealand (HIC)	730	Not reported	730	18 years	3 years	161/730	DSM‐III‐R
Schmid (2011)	Germany (HIC)	314	99%	314	4.5 years	14.5 years^c^	43/314	SCID; BDI; K‐SADS; MEI
Stavrakakis 2013	Netherlands (HIC)	2149	Not reported	2149	(13.02 years)	7.5 years^c^	140/1396	CIDI
Stringaris (2015)	France, UK, Ireland, Germany (HIC)	1554	Not reported	1554	(14.5 years)	2 years	29/999	DAWBA
Wilkinson (2013)	England (HIC)	658	Not reported	658	12–16 years (13.7 years)	1 year	62/598	K‐SADS
Wilkinson (2018)	England (HIC)	945	92%	945	14 years	3 years	68/848	K‐SADS
Wu (2017)	China (UMIC)	386	0%	337	8–17 years; (12.2 years) [2.1]	1 year	33/386	CDI

^a^ High‐income country (HIC); Upper‐middle income country (UMIC); Low‐and middle‐income country (LMIC).

^b^ Authors calculated mean length of follow‐up if study did not report exact length.

^c^ *Instrument abbreviations*: Beck Depression Inventory (BDI); Child and Adolescent Psychiatric Assessment (CAPA); Children's Depression Inventory (CDI); Center for Epidemiology Scale for Depression (CES‐D);World Health Organization Composite International Diagnostic Interview (CIDI); Development and Well‐Being Assessment (DAWBA); Diagnostic Interview Schedule for Children (DISC); Diagnostic and Statistical Manual of Mental Disorders (DSM)‐III‐R; International Classification of Diseases, Tenth Revision (ICD‐10); Kiddie‐Schedule for Affective Disorders and Schizophrenia (K‐SADS); Manheim Parent Interview MEI; Patient Health Questionnaire (PHQ‐9); Expanded Schedule for Affective Disorders and Schizophrenia‐Lifetime (SADS‐L); Structured Clinical Interview for Diagnostic and Statistical Manual‐IV (SCID); Short Moods and Feelings Questionnaire (SMFQ).

The studies featured a wide variation in baseline sample sizes, ranging from 62 to 60 066 young people. Studies that met the inclusion criteria did not include young people above age 19 at baseline; young people’ baseline ages ranged from 4.5 to 19 years across the studies. Table 2 includes the mean age at baseline for studies that included enough information for calculation; however, we cannot report a mean age across studies due to lack of reporting. The estimated length of follow‐up for first‐onset depression across the 19 studies ranged from 9 months to 14.5 years, with a mean of 4.6 years and a median of 3 years. Reporting the exact length of follow‐up varied across studies, where some studies were less clear, and therefore the authors calculated an estimation and included this in Table 2.

Depression was the primary outcome in each study (see Suppl. Table S2). The most common depression assessment instrument was the Kiddie‐Schedule for Affective Disorders and Schizophrenia (K‐SADS). Other instruments included the Structured Clinical Interview for DSM‐IV (SCID), Children's Depression Inventory (CDI), Beck Depression Inventory (BDI), Patient Health Questionnaire (PHQ‐9), Short Moods and Feelings Questionnaire (SMFQ), Child and Adolescent Psychiatric Assessment (CAPA), Composite International Diagnostic Interview (CIDI), Center for Epidemiology Scale for Depression (CES‐D), Development and Well‐Being Assessment (DAWBA), Diagnostic Interview Schedule for Children (DISC), International Classification of Diseases, Tenth Revision (ICD‐10), and the Mannheim Parent Interview MEI.

**FIGURE 2:**
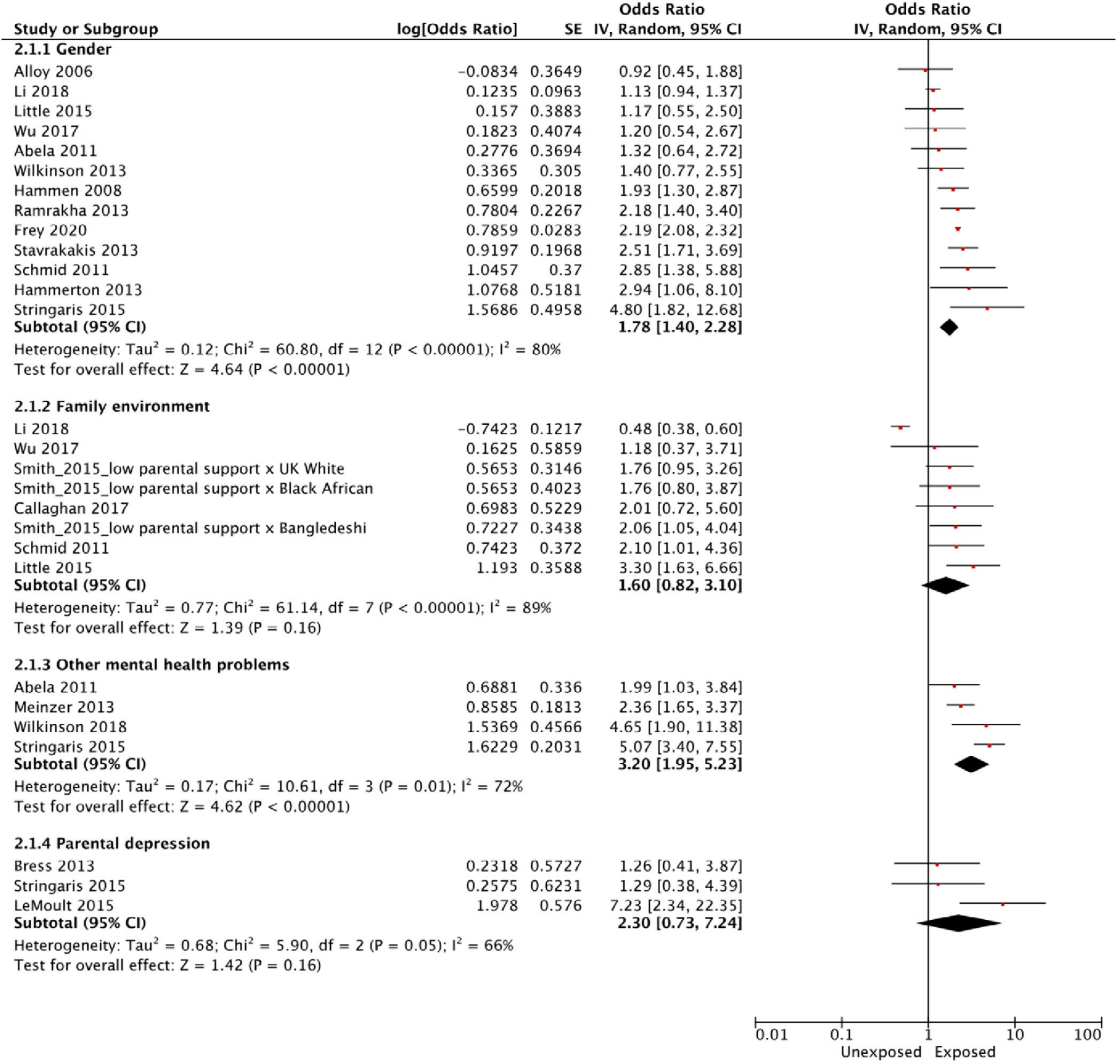
Forest plot of risk factor categories predicting first‐onset depression outcome among study populations (*n* = 19) †Forest plot shows four risk factor categories (gender, family environment, other mental health problems, and parental depression) in which three or more studies were compiled per category. We conducted random‐effects meta‐analyses in Cochrane Review Manager version 5.3 and calculated odds ratios (OR) for binary outcomes with 95% confidence intervals and two‐sided *p*‐values for each risk factor category. Heterogeneity was assessed using the I‐squared statistic within Cochrane Review Manager 5.3. * *Forest Plot Abbreviations*: Chi‐square (Chi^2^); Confidence interval (CI); Degrees of freedom (df); Heterogeneity (*I* ^2^); Interval variable (IV); Standard error (SE)

Prevalence of first‐onset depression, as a percentage of non‐depressed baseline young people with complete data, ranged from 2.90% for a sample size of 999 to 53.23% for a sample size of 62. Details of the study risk factors, risk factor categories, measurement type, and analysis type in relation to first‐onset depression can be found in Table 3. Final risk factor categories for this study included (a) gender, relating to any report of participant gender; (b) family environment, involving any risk factors relating to family relationships and environments; (c) other mental health problems (e.g., ADHD) excluding other mood disorders or symptoms of mood disorders and d) parental depression, relating to any measurement of parental or caregiver depression. Each risk factor category had at least three studies to support the analysis of predicting first‐onset depression. Results are presented as odds ratios (OR), with those exposed to the risk factor being more likely to become depressed (see forest plot in Figure 2). The risk category for gender supports existing literature, where being female leads to a higher risk of depression onset than being male (*n* = 13; OR = 1.78 [1.40, 2.28], *p* < .001, *I* ^2^ = 80%). The gender risk category only represents reported male/female gender categories. None of the studies included reports on how gender data was collected, nor did they report other sex or gender identities or expressions. Young people with other mental health problems at baseline had an increased odds for future first‐onset depression compared to young people that did not have other mental health problems at baseline (*n* = 4; OR = 3.20 [1.95, 5.23], *p* < .001, *I* ^2^ = 72%). There was a non‐significant association with less supportive or dysfunctional family environments contributing to greater odds for future depression compared to young people reporting supportive or functional family environments (*n* = 8; OR = 1.60 [.82, 3.10], *p* = .16, *I* ^2^ = 89%). Similarly, there was a lack of significance for the association of parental history of depression with future onset among their children (*n* = 3; OR = 2.30 [.73, 7.24], *p* = .16, *I* ^2^ = 66%). Heterogeneity ranged from ‘moderate’ to ‘high’ across risk factor categories (Higgins et al., 2003).

**TABLE 3:** Study (*n* = 19) risk factor(s), corresponding risk factor category, risk factor measurement type, analysis type for risk factor relation to first‐onset depression

First author (year)	Risk factor(s)	Risk factor category	Measurement type^a^ (risk factor)	Analysis type for relation to first onset of depression	Adjusted or unadjusted OR
Abela (2011)	Gender; rumination	Gender; other mental health problems	Not reported (gender); CRSQ‐rumination subscale (rumination)	Logistic regression analysis	Adjusted
Alloy (2006)	Gender	Gender	Not reported (gender)	Hierarchical logistic regression	Adjusted
Bress (2013)	Parental depression	Parental depression	Mood module of PHQ‐9 (parental depression)	Authors calculated OR	Unadjusted
Callaghan (2017)	Maternal aggressive behaviour during mother‐adolescent interactions	Family environment	EPI, pleasant events checklist, and issues checklist (maternal aggressive behaviour)	LIFE coding system; Authors calculated OR	Adjusted
Frey (2020)	Gender	Gender	Not reported (gender)	Authors calculated OR	Unadjusted
Hammen (2008)	Gender	Gender	Not reported (gender)	Authors calculated OR	Unadjusted
Hammerton (2013)	Gender	Gender	Not reported (gender)	Authors calculated OR	Unadjusted
LeMoult (2015)	Mothers with current or history of depression	Parental depression	SCID (mothers' current or history of depression)	Authors calculated OR	Unadjusted
Li (2018)	Gender; lives with only one parent	Gender; family environment	Not reported (gender); Not reported (family environment)	Authors calculated OR	Unadjusted
Little (2015)	Gender; maternal aggressive behaviour during mother‐adolescent interactions	Gender; family environment	Not reported (gender); EPI, Pleasant Events Checklist, and Issues Checklist (maternal aggressive behaviour)	LIFE coding system; Authors calculated OR	Unadjusted
Meinzer (2013)	Attention‐deficit/hyperactivity disorder (ADHD)	Other mental health problems	K‐SADS administered to adolescents (ADHD); K‐SADS administered to subset of parents (ADHD)	Authors calculated OR	Unadjusted
Ramrakha (2013)	Gender	Gender	Not reported (gender)	Authors calculated OR	Unadjusted
Schmid (2011)	Gender; mother‐infant interaction—stimulation	Gender; Family environment	Not reported (gender); Categorical System for micro‐Analysis of the Early Mother–Child Interaction (mother‐infant interaction—stimulation)	Logistic and linear regression	Unadjusted for gender; Adjusted for family environment
Smith (2015)	Parental involvement with school: ‘Parental support low‐Bangledeshi’, ‘Parental support low—Black African’, ‘Parental support low—UK White	Family environment	SR (parental support per ethnic population)	Logistic regression analysis	Adjusted
Stavrakakis (2013)	Gender	Gender	Not reported (gender)	Authors calculated OR	Unadjusted
Stringaris (2015)	Gender; Any conduct disorder; Family history of depression	Gender; Other mental health problems; Parental depression	Not reported (gender); SDQ (any conduct disorder); Not reported (family history of depression)	Authors calculated OR	Unadjusted
Wilkinson (2013)	Gender	Gender	Not reported (gender)	Multiple logistic regression	Adjusted
Wilkinson (2018)	Non‐suicidal self‐injury (NSSI)	Other mental health problems	Binary SR (NSSI); self‐report of frequency (NSSI)	Logistic regression analysis	Adjusted
Wu (2017)	Gender; Family functioning	Gender; Family environment	Not reported (gender); APGAR (family functioning)	Authors recalculated OR^b^	Adjusted

^a^ Measurement Abbreviations: Attention Deficit/Hyperactivity Disorder (ADHD); Adaptation Partnership Growth Affection Resolve‐Family (APGAR); Children's Response Styles Questionnaire (CRSQ)‐Rumination subscale; Emotionality Activity Sociability (EAS) Questionnaire; Event Planning Interaction (EPI); The Living In Family Environments (LIFE); Non‐suicidal Self‐Injury (NSSI); Odds Ratio (OR); Patient Health Questionnaire (PHQ‐9); Structured Clinical Interview for Diagnostic and Statistical Manual (SCID); Strengths and Difficulties Questionnaire (SDQ); Self‐reported (SR).

^b^ Family functioning was reported as a protective factor, and authors recalculated odds ratio to report as risk factor.

We used SAQOR quality assessment criteria with modified GRADE rankings (Ross et al., 2011) (see Table 4). Ten studies were marked ‘Adequate’ across four or more of the six SAQOR categories and remained at the initial GRADE rating for observational studies of ‘low’ quality. The other nine studies had three or more categories marked as ‘Inadequate’ and were downgraded to a GRADE rating of “very low” (Guyatt et al., 2011). Four out of the six SAQOR domains were typically not fully met. Papers typically did not meet one or more criteria in four of the six SAQOR domains. In *quality in measurement*, 37% of studies did not meet full criteria, such as authors not clearly defining the tools or methods used to measure the study outcome. In *reporting on follow‐up*, 58% of studies did not meet full criteria, in some cases due to authors' not providing an explanation for how or why study participants were not followed‐up on. In *reporting of distorting influences*, 53% of studies did not meet one or more criteria, for reasons such as not accounting for other psychiatric comorbidities during analysis, lack of explanation for how other treatment the participants may have been taking was dealt with, or what and how potential confounders were considered and assessed. In *reporting missing data or an unclear presentation of data*, 63% of papers failed to meet all criteria. For example, authors may not have explained how missing data was addressed or did not clearly and accurately present data (e.g., all data does not add up and other confusions regarding any data presented).

**TABLE 4:** Systematic assessment of quality in observational research (SAQOR) quality assessment ratings of included studies (*n* = 19)

First author (year)	Sample	Control/comparison group	Measurement quality	Follow‐up	Distorting influences	Reporting of data	Modified grade rating
Abela (2011)	Adequate	Adequate	Inadequate	Inadequate	Inadequate	Inadequate	Very low
Alloy (2006)	Adequate	Adequate	Adequate	Inadequate	Adequate	Inadequate	Low
Bress (2013)	Adequate	Adequate	Adequate	Adequate	Inadequate	Adequate	Low
Callaghan (2017)	Adequate	Inadequate	Adequate	Inadequate	Adequate	Inadequate	Very low
Frey (2020)	Adequate	Adequate	Adequate	Adequate	Inadequate	Inadequate	Low
Hammen (2008)	Adequate	Adequate	Adequate	Adequate	Inadequate	Inadequate	Low
Hammerton (2013)	Adequate	Adequate	Adequate	Inadequate	Adequate	Adequate	Low
LeMoult (2015)	Adequate	Adequate	Adequate	Adequate	Inadequate	Inadequate	Low
Li (2018)	Adequate	Adequate	Inadequate	Adequate	Inadequate	Inadequate	Very low
Little (2015)	Adequate	Adequate	Adequate	Adequate	Inadequate	Adequate	Low
Meinzer (2013)	Inadequate	Adequate	Adequate	Adequate	Adequate	Adequate	Low
Ramrakha (2013)	Inadequate	Adequate	Adequate	Inadequate	Adequate	Inadequate	Very low
Schmid (2011)	Adequate	Adequate	Adequate	Adequate	Adequate	Inadequate	Low
Smith (2015)	Adequate	Adequate	Inadequate	Inadequate	Inadequate	Adequate	Very low
Stavrakakis (2013)	Adequate	Adequate	Inadequate	Inadequate	Inadequate	Adequate	Very low
Stringaris (2015)	Adequate	Adequate	Adequate	Inadequate	Adequate	Inadequate	Low
Wilkinson (2013)	Inadequate	Inadequate	Inadequate	Inadequate	Adequate	Adequate	Very low
Wilkinson (2018)	Adequate	Adequate	Inadequate	Inadequate	Inadequate	Inadequate	Very low
Wu (2017)	Adequate	Adequate	Inadequate	Inadequate	Adequate	Inadequate	Very low

## 4 DISCUSSION

In this meta‐analysis, we identified 19 prospective studies representing 21 unique populations that identified risk factors predicting first‐onset depression among young people aged 10–25. Heterogeneity was high across all analyses conducted, for example, *I* ^2^ > 70%. Given the small number of studies for each risk factor, it was not possible to do subgroup analyses to determine sources of heterogeneity. Likely reasons for heterogeneity include range of age at baseline (4.5–19 years), diversity of tools used to assess depression (13 tools across 19 studies), diversity of how risk factors were operationalized (self‐report vs. objective records), and how risk exposure was assessed. For example, there was a lack of consistency across studies in this review about when exposure to the risk factor occurred (e.g., interaction with aggressive maternal behaviour), and of the included studies, the mean length of follow‐up was approximately 4.6 years, potentially offering too large a window for unrecorded confounding risk factors to affect first‐onset depression. Risk factors in this meta‐analysis (categories including gender, family environment, other mental health problems, and parental depression) relate to the existing evidence base, particularly among studies conducted in high‐income settings. For example, mental health problems (e.g., prior anxiety and distress disorders) and gender have been known risk factors for onset of depression, with the female gender showing to substantially increase the risk in early to mid‐adolescence (Hankin et al., 1998; Salk et al., 2017).

Other previously reported risk factors were not found to be statistically associated with future first‐onset depression in this analysis. For example, related to the family environment (mothers' aggressive behaviour, living with only one parent, low family support, and low family functioning), a systematic review and meta‐analysis (Yap et al., 2014) identified parental conflict, less warmth from parents, parental aversiveness, and parental monitoring as risk factors for both anxiety and depression among 12–18‐year‐olds. The authors of this meta‐analysis combined results from longitudinal, retrospective and cross‐sectional studies, and included both symptoms and diagnoses of anxiety or depression as outcome variables combatively. These methodological approaches could support a significant finding, and both approaches differ from those taken in our analysis. Similarly, parental depression, particularly maternal depression, has been increasingly assessed as a risk factor for adolescent depression; a systematic review and meta‐analysis on the risk of depression in adolescent offspring of mothers who experienced perinatal depression, found an increased odds of depression (pooled OR, 1.70, 1.60–2.65), with subgroup analyses finding slightly higher odds (78% increase) for antenatal studies compared to postnatal studies (66% increase) (Tirumalaraju et al., 2020). It is possible this study may have identified a significant association due to the authors' focus on perinatal depression only and use of a specific timepoint for assessment of perinatal depression (1 year pre‐ or post‐birth), whereas our study included measures of parental depression assessed at any time and with either parent. Additionally, our review controlled for assessment of depression at baseline and required that follow‐up depression assessment be between 10 and 25 years old in order to measure first‐onset of depression for adolescents and young people.

We could not identify a broad range of risk factors predicting first‐onset depression across diverse populations, particularly among LMICs, in this review. However, similar risk factors have been identified across various global settings and cultures in other observational study types. For example, young age and depression are consistently reported as risk factors for suicidal thought and behaviours as well as family conflicts, other mental health or substance use disorders, and socioeconomic factors in South Asia (Kohrt et al., 2012; Marahatta et al., 2017; Upadhaya et al., 2019). Also, prevalence of depression and incidence of suicide are highest among females under age 25 in this region (Hagaman et al., 2017; Luitel et al., 2013).

Of the existing cohorts in HIC and LMICs which have collected data on risk factors for first‐onset depression, only a few have published results that represent participants without depression at baseline. Among the full‐text articles we excluded for lack of analysis for first‐onset depression in this study, at least 34% (112 out of 325) resulted from not reporting or controlling for baseline or history of MDD. Those that have access to these dataset types are encouraged to publish these findings.

A number of well‐known longitudinal studies were not included in the current analyses, for example, the Dunedin Multidisciplinary Health and Development Study (Beckley et al., 2021; Dunedin Multidisciplinary Health & Development Research Unit, 2022), Oregon Adolescent Depression Project (Oregon Research Institute, 2013), the Weissman 30‐year generational cohort (Weissman et al., 2016), Zurich Cohort Study (Angst et al., 1984; Angst et al., 2005), Early Developmental Stages of Psychopathology (EDSP) (Wittchen et al., 1998), and Christchurch Health and Development Study (Fergusson & Horwood, 2001). The majority of these studies were excluded in the full‐text stage due to lack of clear reporting for first‐onset of depression, with the exception of one EDSP study (Beesdo et al., 2007), which was excluded due to being published outside of our included date range. Common reasons for exclusion were not controlling for baseline depression, combining depression outcomes for those with and without a history of depression, or age range issues such as combining results for age ranges below and above 25. For instance, the EDSP cohort data was analysed for negative emotionality as a risk factor associated with depression at age 18 but did not report a measurement of depression at baseline or any point before age 18 (Bould et al., 2014). As a result, depression diagnoses at follow‐up did not meet our first‐onset depression outcome criteria. Another analysis of EDSP cohort data examined incidence, comorbidity, and risk patterns for anxiety and depressive disorders (Beesdo et al., 2010). However, the data presented for onset of depressive disorder was a cumulative age of 34, and we could not extract onset for depression within our included age range (10–25 years). An analysis from the Oregon cohort data was evaluated for the relationship between childhood respiratory symptoms and later depression but did not distinguish between those with and without a history of depression (Goodwin et al., 2013). In this case, combining depression outcomes for those with and without a history of depression prevented the determination of a clear link between the risk factor and first‐onset depression. Analyses using data from the Christchurch cohort reported pooled results for onset of MDD for age groups 18, 21, 25 and 30, and therefore we could not include these in our study (Fergusson et al., 2014; Fergusson, Boden, & Horwood, 2013; Fergusson, McLeod, & Horwood, 2013; Fergusson, McLeod, & Horwood, 2015; Fergusson, McLeod, Horwood, Swain, et al., 2015; Newton‐Howes et al., 2015).

Overall, due to the heterogeneity in design, analyses, and reporting of data in major longitudinal studies, it was not possible to draw upon many of the large cohort studies for the current meta‐analysis. Therefore, to inform prevention programs and increase accurate detection of risk and protective factors for first‐onset depression among young people globally, we offer recommendations for future research and analyses in Box 1.

Box 1 **Recommendations for study design, analysis and reporting for longitudinal studies of first‐onset depression among adolescents and young people**

1. Populations studied: Include more racially, ethnically and globally representative samples in longitudinal adolescent depression research.
  Youth living in LMICs experience the highest risk for first‐onset depression and suicide due to their environmental and social circumstances, such as increased rates of poverty, violence, natural disasters and lack of available psychological treatments (Belfer, 2008; Erskine et al., 2015; Kieling et al., 2011; Leckman & Leventhal, 2008; WHO, 2014). Future research must be more representative of the global adolescent population and conduct longitudinal studies that identify common and unique contextual risk factors across a variety of globally representative populations. For example, the Identifying Depression Early in Adolescence (IDEA) consortium has analyzed longitudinal samples for constellations of risk factors in diverse populations including Brazil, Nigeria, and Nepal (Brathwaite, Rocha, Kieling, Gautam, et al., 2021; Brathwaite, Rocha, Kieling, Kohrt, et al., 2020; Rocha et al., 2021)
2. Sample selection: Longitudinal study designs with young people should clearly recruit, confirm, and report on subsamples without current or prior depression at baseline.
  To identify risk factors for first‐onset depression among young people, it is crucial to begin with a population without current or prior depression. A history of depression confounds the exploration of potential causal risk factors in longitudinal studies. For example, longitudinal studies on the role of substance use or physical inactivity need to begin with a depression‐free cohort to demonstrate prospectively how these risk factors may play a potentially causal role. Similarly, if studies on parental depression do not have a depression‐free sample at baseline, the cause could be misconstrued, for example, the parental and adolescent depression could have had a common cause, or the adolescent depression could precede the parental depression. Ultimately, if participants are depressed at baseline, risk factors may be correlates or secondary outcomes of depression rather than risk factors.
3. Validated assessment of depression: Instruments used in longitudinal studies should be validated for both the age ranges in the study as well as for the specific linguistic and cultural groups participating.
  For studies reporting outcomes with continuous measures for depression (e.g., PHQ‐9/PHQ‐A, CDI and BDI), we recommend the researchers validate the tool(s)—including the designated cut‐off point for depression diagnosis—within the given population, accounting for language, developmental age, and cultural patterns of responding to mental health assessments (Kohrt et al., 2011). Initiatives such as UNICEF's Measurement of Mental Health among Adolescents at the Population Level (MMAP) can be a source of locally and developmentally validated tools for depression research (Carvajal‐Velez et al., 2021). Additionally, future researchers should avoid combining other disorder outcomes, such as bipolar or anxiety disorder, with first‐onset depression, to better determine specific risk factors for unipolar depression.
4. Operationalization of risk factor measurement: Common definitions, measurement strategies, and time points are needed for risk factors.
  Harmonization of how risk and protective factors are operationalized and measured will enhance the ability to conduct cross‐cultural data analysis, including testing prediction models and measurement of at‐risk young people from multiple data sets worldwide (Brathwaite, Rocha, Kieling, Gautam, et al., 2021). For example, for the risk factor, ‘family environment’, included studies assessed maternal aggressive parenting behaviours (Callaghan et al., 2017; Little et al., 2015), mother‐infant interaction (Schmid et al., 2011), report of parent involvement with school (Smith et al., 2015), report of living with only one parent (Li et al., 2018), and satisfaction of perceived family functioning (Wu et al., 2017). Studies in the gender category lacked clarity in measurement: most included a self‐report (male/female) but did not decipher gender as a biological variable (defined by DNA encoded, physiological characteristics). Future research could account for how the male/female or other gender categorization was determined and its potential effects on outcomes, as recommended by the U.S. National Institutes of Health (National Institute for Health, 2017). Moreover, the timing of exposure to social and environmental risk should be consistently recorded and reported.
5. Use of multiple risk factors: Studies should assess multiple risk factors and consider development of risk factor scores.
  Risk factors should not be studied in isolation. The epidemiology and psychiatry fields have established the increasing importance of using multicausal models for illnesses; yet most risk factors for psychiatric illnesses continue to be studied in isolation (Hill, 1965; Kendler, 2019). Rather than attempting a reductionist approach to determine a single cause that is ‘necessary and sufficient’, researchers should identify a ‘web of causation’ (Kendler, 2019; Krieger, 1994) and assess multiple interactions of multiple causes to identify pathways that predict the onset of depression, including both risk and protective factors. Regardless of whether it is adequate or essential, blocking a component factor or cause of first‐onset depression offers the ability to prevent onset of depression (Kendler, 2019; Rothman & Greenland, 2005). The IDEA consortium studies have used a constellation of 11 risk factors to calculate future depression with greater than chance accuracy (Brathwaite, Rocha, Kieling, Gautam, et al., 2021; Brathwaite, Rocha, Kieling, Kohrt, et al., 2020; Rocha et al., 2021).

### 4.1 Limitations

Our study has several limitations. Due to the small number of studies included across most of the risk factor categories, we cannot uncover sources of heterogeneity across studies or run tests for asymmetry. Also, we are unable to address additional risk factors that have been previously shown to increase risk of depression. For example, we attempted to include risk categories that covered substance use (e.g., smoking and alcohol use) and physical health problems (e.g., asthma, body mass index and blood pressure). We identified only two studies that were initially eligible but then excluded for not meeting criteria to substantiate a risk factor category in our analyses (Chen et al., 2014; Hammerton et al., 2013). Due to the lack of evidence, this review was unable to address or control for protective factors. Finally, this review focused on identifying exposures to psychological and contextual factors that could lead to a depression outcome, rather than assessing the effects of an intervention (e.g., preventative interventions) on a depression outcome. Given the absence of preventive interventions in this review, it is not possible to make any GRADE classification of recommendations for intervention strategies. A recent review has summarized the prevention literature for young people (Fusar‐Poli et al., 2021).

## 5 CONCLUSION

This systematic review and meta‐analysis strengthen evidence on risk factors predicting first‐onset depression in adolescence and young people, revealing longitudinal prospective effects for gender and other mental health problems in high‐income and one upper‐middle‐income countries. We were unable to identify any longitudinal studies that further support evidence for these risk factors in LMIC. Based on common shortcomings of the current literature, we provided five recommendations for future research on risk factors for first‐onset depression among young people: first, include more ethnically, racially, and diverse populations; second, in recruitment and analysis, clearly identify the subsample of participants who had no current or prior depression at baseline assessment; third, validate the depression assessment within the given study population for both the age ranges and the specific linguistic and cultural groups participating; fourth, during protective and risk factor measurement, use common definitions, measurement strategies, and data collection time points to promote conduct of cross‐cultural data analysis; fifth, assess multiple risk and protective factors, and consider development of risk factor scores, to identify pathways for predicting the onset of depression. Reducing the incidence of depression is a global health necessity, and preventive strategies have shown promising results (Cuijpers et al., 2012). To support the prevention of first‐onset depression in young people, future research could develop strategies that give greater attention to the strongest risk factors consistently evidenced in the literature (Ormel et al., 2019), such as focusing on the mental health needs of children that identify as female, or children with psychological comorbidities. In addition, prevention strategies could target a constellation of risk factors and go beyond the individual or relationship level. Prevention strategies taking place in local institutions (e.g., schools) have been found to be acceptable, increase reach, allow for modular approaches that can be tailored to the individual or group, and increase the chance for sustainability (Fazel & Kohrt, 2019; Ormel et al., 2019; Weist et al., 2019). A larger scope of risk and protective factors for first‐onset depression must be identified globally to inform best prevention strategies across settings.

## CONFLICT OF INTEREST

VM has received research funding from Johnson & Johnson, a pharmaceutical company interested in the development of anti‐inflammatory strategies for depression, but the research described in this paper is unrelated to this funding. All other authors declare that they have no competing interests.

## AUTHORS CONTRIBUTIONS

BAK and CK conceived the idea for the review. GAP, BAK, CK, HLF, VM, and ZZ developed the review protocol. GAP, KG, CL, and MH conducted the search with supervision by BAK. CL, MH and GAP performed extraction. GAP and BAK did the statistical analysis. GAP and BAK wrote the first draft of the manuscript with input from CL and MH. All authors critically appraised and edited the manuscript. All authors contributed to revision and finalization of the manuscript.

## Supporting information


**Supplemental Figure S1** Titles and abstracts screening and related interrater reliability (IRR) processes

**Appendix** **S1**: Supporting Information

## Data Availability

Data sharing is not applicable to this article as no new data were created or analyzed in this study.
