# Supplementary figures and images for: Psychological and contextual risk factors for first‐onset depression among adolescents and young people around the globe: A systematic review and meta‐analysis
Source: Early Interv Psychiatry. 2022 Apr 7;17(1):5–20. doi: 10.1111/eip.13300 (PMC10084304; doi:10.1111/eip.13300)

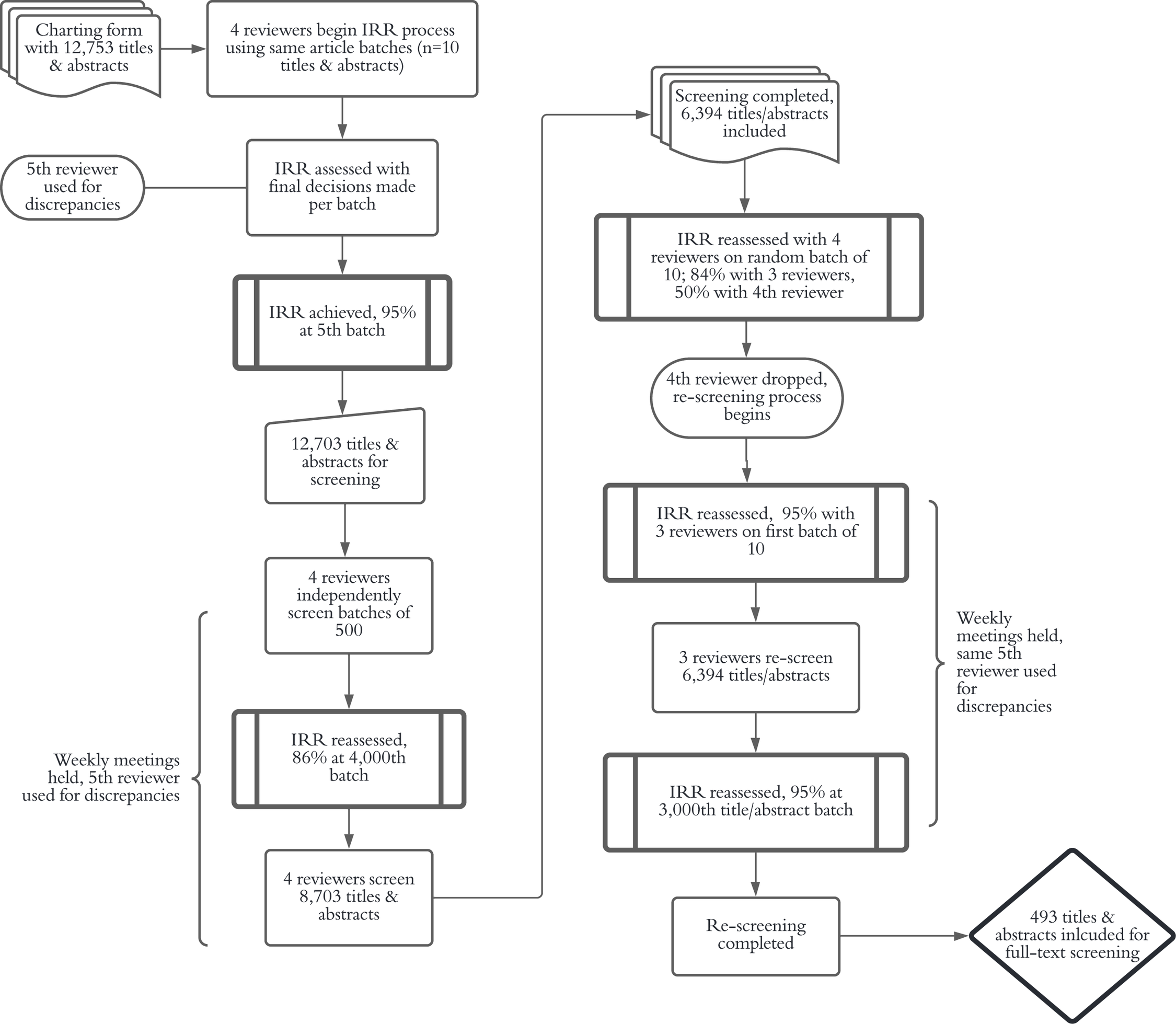

Supplement: Supplementary file 1 — Supplemental Figure S1 Titles and abstracts screening and related interrater reliability (IRR) processes [file EIP-17-5-s001.tif]
