# Supplementary material for: Psychological and contextual risk factors for first‐onset depression among adolescents and young people around the globe: A systematic review and meta‐analysis
Source: Early Interv Psychiatry. 2022 Apr 7;17(1):5–20. doi: 10.1111/eip.13300 (PMC10084304; doi:10.1111/eip.13300)
Supplement: Supplementary file 2 — Appendix S1: Supporting Information [file EIP-17-5-s002.docx]

| Supplemental Box S1. Sample search strategy for Medline (Ovid) used for systematic review |
| --- |
| 1. ADOLESCENT/ |
| 2. (adolesc* or "youth" or "teen" or "young people" or "young person*").mp. [mp=title, abstract, original title, name of substance word, subject heading word, floating sub-heading word, keyword heading word, protocol supplementary concept word, rare disease supplementary concept word, unique identifier, synonyms] |
| 3. Young Adult/ |
| 4. 1 or 2 or 3 |
| 5. DEPRESSION/ |
| 6. Depressive Disorder/ |
| 7. Depressive Disorder, Major/ |
| 8. ("depressive disorder" or "symptoms of depression" or "major depressive disorder").mp. [mp=title, abstract, original title, name of substance word, subject heading word, floating sub-heading word, keyword heading word, protocol supplementary concept word, rare disease supplementary concept word, unique identifier, synonyms] |
| 9. 5 or 6 or 7 or 8 |
| 10. LONGITUDINAL STUDIES/ |
| 11. Prospective Studies/ |
| 12. Observational Study/ |
| 13. (longitudinal or prospective or "observational stud*").mp. [mp=title, abstract, original title, name of substance word, subject heading word, floating sub-heading word, keyword heading word, protocol supplementary concept word, rare disease supplementary concept word, unique identifier, synonyms] |
| 14. 10 or 11 or 12 or 13 |
| 15. 4 and 9 and 14 |

**Supplemental Figure S1. Titles and abstracts screening and related interrater reliability (IRR) processes**

Process diagram showing the process for screening titles and abstracts and assessing interrater reliability (IRR). Stages include: 1. Charting form with titles and abstracts and number of reviewers for screening, 2. IRR process and achievement; 3. Independent screening with weekly meetings for discussions and use of an outside reviewer for discrepancies; 4. Reassessment of IRR and continuance of independent screening with weekly meetings for discussions and use of an outside reviewer for discrepancies; 5. Number of titles and abstracts included after first round of all titles and abstracts screened; 6. Reassessment of IRR with four reviewers and then with three reviewers; 7. Decision to drop fourth reviewer; 8. Reassessment of IRR with 3 reviewers and IRR achieved; 10. Independent re-screening of 6,394 titles and abstracts with weekly meetings for discussions and use of an outside reviewer for discrepancies; 11. Reassessment of IRR and achievement; 12. Screening completed with number of titles and abstracts inckuded for full-text screening.

**Supplemental Table S1:** Screening criteria in 4x4 table for ascertaining first-onset depression for study inclusion

|  | First-onset depression at follow-up | No onset of depression at follow-up |
| --- | --- | --- |
| **With risk factor at baseline** | # of participants with:   1. no history or current depression at baseline 2. risk factor at baseline 3. first-onset MDD†/ depression at follow-up | # of participants with:   1. no history or current depression at baseline 2. risk factor at baseline 3. no MDD/ depression at follow-up |
| **Without risk factor at baseline** | # of participants with:   1. no history or current depression at baseline 2. no risk factor at baseline 3. first-onset MDD/ depression at follow-up | # of participants with:   1. no history or current depression at baseline 2. no risk factor at baseline 3. no MDD/ depression at follow-up |

†*Abbreviations*: Major depressive disorder (MDD)

**Supplemental Table S2**: Measurement instruments for first-onset depression in adolescents in included studies (*n*=19)

| First Author (Year) | Primary Outcome | Measurement Instrument † | Additional Measures |
| --- | --- | --- | --- |
| Abela (2011) | Depression | K-SADS | CDI |
| Alloy (2006) | Depression | SADS-L | BDI |
| Bress (2013) | Depression | PHQ-9 | DISC |
| Callaghan (2017) | Depression | K-SADS-PL | N/A |
| Frey (2020) | Depression | ICD-10 | N/A |
| Hammen (2008) | Depression | K-SADS-E; SCID | N/A |
| Hammerton (2013) | Depression | CAPA | N/A |
| LeMoult (2015) | Depression | K-SADS-P; SCID | CDI-S |
| Li (2018) | Depression | CES-D | N/A |
| Little (2015) | Depression | K-SADS-PL | N/A |
| Meinzer (2013) | Depression | K-SADS; SCID-NP | N/A |
| Ramrakha (2013) | Depression | DSM-III-R; DSM-IV | N/A |
| Schmid (2011) | Lifetime diagnosis for any depressive disorder | MEI; K-SADS-PL; SCID | BDI |
| Smith (2015) | Depression and mental well-being. | SMFQ | N/A |
| Stavrakakis (2013) | Depression | CIDI | N/A |
| Stringaris (2015) | Depression and anhedonia | DAWBA | N/A |
| Wilkinson (2013) | Depression | K-SADS-L | MFQ |
| Wilkinson (2018) | Depression and other psychiatric disorders (anxiety, eating disorders, behaviour disorders, substance use disorder, alcohol use disorder) | K-SADS-PL | MFQ |
| Wu (2017) | Depression | CDI | N/A |

† *Instrument abbreviations:* Beck Depression Inventory (BDI); Child and Adolescent Psychiatric Assessment (CAPA); Children’s Depression Inventory (CDI); Center for Epidemiology Scale for Depression (CES-D); World Health Organization Composite International Diagnostic Interview (CIDI); Development and Well-Being Assessment (DAWBA); Diagnostic Interview Schedule for Children (DISC); Diagnostic and Statistical Manual of Mental Disorders (DSM)-III-R; International Classification of Diseases, Tenth Revision (ICD-10); Kiddie-Schedule for Affective Disorders and Schizophrenia (K-SADS); Manheim Parent Interview MEI; Patient Health Questionnaire (PHQ-9); Expanded Schedule for Affective Disorders and Schizophrenia-Lifetime (SADS-L); Structured Clinical Interview for Diagnostic and Statistical Manual-IV (SCID); Short Moods and Feelings Questionnaire (SMFQ)

**SUPPLEMENTAL TABLE LEGEND**

**Table S2:** Measurement instruments for first onset depression in adolescents in included studies (*n*=19)

The table shows the instruments used to measure depression at baseline and follow-up for young people in included studies. In some studies, researchers administered the instruments to both young people and their parents. The most common instruments used were variations of the Kiddie-Schedule for Affective Disorders and Schizophrenia (K-SADS). Additional measures often included self-report instruments to supplement the primary measurement of depression.
